# Curative Effect and Autonomic Nerve Function of Patients With Primary Insomnia of Liver Depression and Spleen Deficiency Type Based on the Acupoint Selection of Meridian Theory: Protocol for a Randomized Controlled Trial

**DOI:** 10.2196/84122

**Published:** 2026-01-22

**Authors:** Su Fu, Kang He, Chuanlong Zhou

**Affiliations:** 1 Department of Acupuncture and Moxibustion, The Third Affiliated Hospital of Zhejiang Chinese Medical University, 219 Moganshan Road, Hangzhou, Zhejiang, 310005, China, 86 15868486255

**Keywords:** liver stagnation and spleen deficiency insomnia, acupuncture, randomized controlled trial, autonomic nerve function, Jing Bie theory, traditional Chinese medicine

## Abstract

**Background:**

Primary insomnia often corresponds to the syndrome of liver depression and spleen deficiency in traditional Chinese medicine. This study evaluates a new acupuncture protocol derived from the “Jing Bie” theory. Tianrong (TE16) and Tianyou (SI17) were selected to regulate the Shaoyang meridian, soothe the liver, regulate qi (the basic life energy or life force to maintain the physiological function of the body according to traditional Chinese medicine), strengthen the spleen, and tranquilize the mind, so as to restore sleep.

**Objective:**

The objective of this study is to evaluate the clinical efficacy of this regimen and to explore its effects on autonomic nervous system function and central nervous system chemistry.

**Methods:**

This is a single-blind randomized controlled trial. A total of 96 patients with primary insomnia (liver depression and spleen deficiency pattern) will be recruited and randomly assigned to the treatment group or the control group. At the same time, 48 healthy volunteers will be recruited as the healthy control group.

**Results:**

This study was funded in November 2023. Recruitment and data collection began in January 2024 and are currently underway. As of December 2025, a total of 40 participants have been enrolled, of whom 4 have withdrawn. Recruitment is projected to conclude by December 2026. Data analysis will be performed after the completion of recruitment. The results are expected to be published in summer 2027.

**Conclusions:**

This study integrates a traditional Chinese medicine framework with modern physiological measurements. The aim is to provide evidence for targeted acupuncture strategies by linking clinical improvement to autonomic nervous system balance and neurochemical changes and to elucidate potential mechanisms to provide a nondrug treatment option for insomnia.

## Introduction

Insomnia is a subjective experience in which patients are not satisfied with the length and/or quality of sleep, and it affects their daytime social function [1]. Long-term insomnia can lead to fatigue, mood swings, and cognitive impairment [2]. Studies have shown that acupuncture is effective in treating insomnia [3]. “Jing Bie” is a longitudinal branch of the 12 meridians, which goes deep into the body cavity to strengthen the connection between the 2 meridians in the deep part of the trunk. The theory of Jing Bie is recorded in “Lingshu · Jing Bie,” and the distribution characteristics of Jing Bie are “leaving, entering, leaving and combining.” Six acupoints, such as Tianrong (TE16) and Tianyou (SI17), are located in the neck [4]. In the theory of traditional Chinese medicine (TCM), Jing Bie not only strengthens the close relationship between the limbs and the viscera, the head, and the 5 sense organs but also plays a role in connecting the 2 meridians, reconciling the “ying” and “wei,” and promoting the balance of qi (the basic life energy or life force to maintain the physiological function of the body) and blood in the head [5]. From the perspective of modern medical anatomy, the 6 acupoints on the neck are mostly located near the sternocleidomastoid muscle, where the superior cervical ganglion and stellate ganglion of the sympathetic trunk are deeply distributed. Intervention in these parts can regulate sympathetic and parasympathetic nerve function and relieve symptoms related to autonomic nervous system (ANS) disorders. For example, stellate ganglion block (SGB) is often used clinically to treat sympathetic-parasympathetic imbalances such as migraine, cluster headache, and insomnia [6,7]. SGB can inhibit central and peripheral activity; jointly control the glands, blood vessels, and muscle movement in the region; and regulate sympathetic nerve activity from a pathologically hyperactive state to normal levels and maintain stability. Repeated SGB can interfere with autonomic nerve activity, thereby modifying autonomic nerve function [8,9]. Therefore, the acupoints selected based on the Jing Bie theory may also have a good effect on autonomic nerve regulation because they are anatomically adjacent to the stellate ganglion.

A study that analyzed the effects of acupuncture on the ANS showed that acupuncture can reduce sympathetic nerve activity under stress conditions and balance the ANS [10]. In a review that included 14 studies on the effects of acupuncture on heart rate variability (HRV), it was reported that acupuncture can reduce the low-frequency power (LF) and the LF to high-frequency power (HF) ratio (LF/HF) in patients and may function to balance the sympathetic and parasympathetic nervous systems [11]. A previous study by the project leader found that acupuncture at the acupoints Tianrong (TE16) and Fengchi (GB20) can improve the autonomic nerve function of patients with migraine. Although studies have shown that acupuncture has a regulatory effect on autonomic nerves, there is still a lack of in-depth discussion on the mechanism [12]. At present, most studies on acupuncture treatment of insomnia do not strictly distinguish TCM syndromes, and it is difficult to accurately provide effective treatment options for specific syndromes. This study focuses on insomnia of liver depression and spleen deficiency type. It is expected to clarify the curative effect of acupuncture on insomnia of this type through rigorous randomized controlled trials (RCTs) and provide more targeted evidence for clinical treatment with TCM.

HRV reflects the functional status of sympathetic and parasympathetic nerves, which offers a window into the functional status of human ANS [13]. Therefore, it is believed that HRV reflects the mechanism of excessive arousal from the changes in autonomic nerve function, and improving HRV indices and reducing excessive arousal may be a new idea for the treatment of insomnia. We designed this study based on the theory of Jing Bie selection to explore the treatment of insomnia through the analysis of HRV.

In “Huangdi Neijing,” there is a discussion on the disharmony between ying and wei in insomnia. According to the theory of branches of 12 meridians, selection of acupoints on the neck can regulate the qi of the 2 meridians; the theory also explains the principle of ANS intervention from the perspective of modern medicine. Acupuncture intervention can regulate autonomic nerve function. In a preliminary study on autonomic nerve intervention in migraine, we found that acupuncture at neck acupoints has a certain therapeutic effect. In modern medical research, HRV, which reflects autonomic nerve function, can well reflect the clinical situation of patients with primary insomnia-hyperarousal, and it is believed that primary insomnia can be treated by improving HRV indices. Therefore, in this study, we aim to examine whether, according to the theory of meridian acupoint intervention, the curative effect of the treatment of primary insomnia can be improved through autonomic nerve intervention.

## Methods

### Study Setting and Design

This study is a single-center, prospective, single-blind RCT. A total of 96 patients with primary insomnia of liver depression and spleen deficiency type will be recruited from the Department of Acupuncture and Moxibustion of the Third Affiliated Hospital of Zhejiang Chinese Medical University in Zhejiang province, China. Participants will be randomly assigned (1:1) to the acupuncture group based on the theory of Jing Bie selection or the conventional acupuncture control group. The intervention will be carried out for 4 weeks, followed by follow-up.

**Figure 1.**
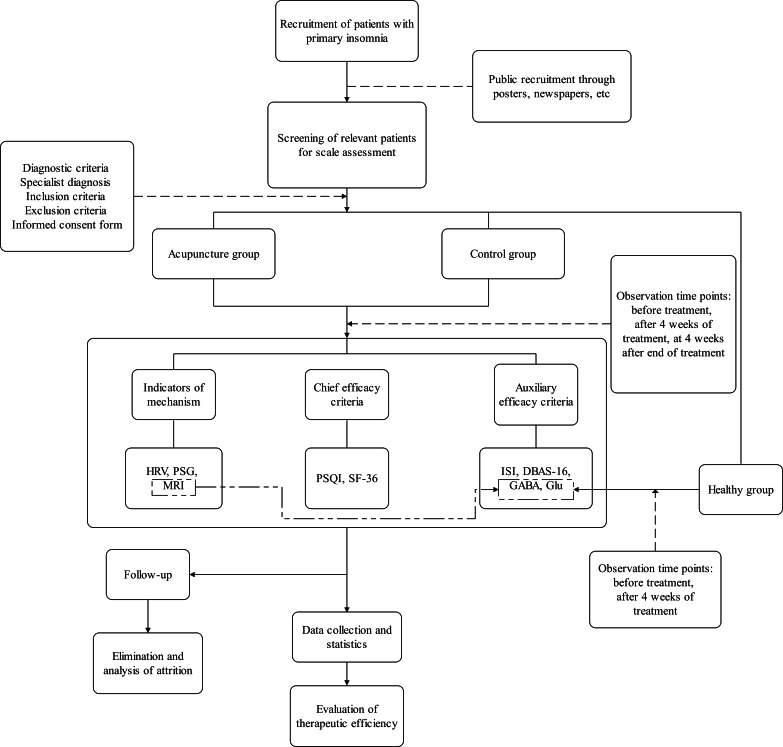
Study flowchart. DBAS-16: 16-item Dysfunctional Beliefs and Attitudes About Sleep scale, GABA: γ-aminobutyric acid, Glu: glutamic acid; HRV: heart rate variability; ISI: Insomnia Severity Index; MRI: magnetic resonance imaging; PSG: polysomnography; PSQI: Pittsburgh Sleep Quality Index; SF-36: 36-item Short Form Health Survey.

The specific follow-up arrangement will be evaluated in the fourth week of the treatment period, and the patients’ recovery and treatment efficacy will be assessed in depth at key follow-up time points. In both groups, the treatment will be administered 3 times a week for 4 weeks. At the same time, 48 healthy volunteers without sleep disorders will be included as the healthy control group without any intervention measures, which will be used to compare and analyze the changes in relevant indicators to evaluate the effect of acupuncture treatment more comprehensively. The flowchart of the study process is shown in Figure 1, and the trial schedule of enrollment, treatments, and assessments is displayed in Table 1. The reporting of this study protocol conforms to the SPIRIT (Standard Protocol Items: Recommendations for Interventional Trials) guidelines (Checklist 1).

**Table 1.** Schedule of study assessments and outcome measures.

Research stage	Before treatment	Treatment stage	Follow-up stage
Assessment	First	Second	Third
Time (weeks)	0	4	8
Informed consent	✓	—^a^	—
Inclusion/exclusion criteria	✓	—	—
Demographic data	✓	—	—
Vital signs	✓	—	—
Medical history record	✓	—	—
Physical examination	✓	—	—
Medication situation	✓	—	—
Indicator of mechanism			
HRV^b^	✓	✓	✓
PSG^c^	✓	✓	✓
MRI^d^	✓	✓	—
Primary outcome indicator			
PSQI^e^	✓	✓	✓
SF-36^f^	✓	✓	✓
Secondary outcome indicator			
ISI^g^	✓	✓	✓
DBAS-16^h^	✓	✓	✓
GABA^i^	✓	✓	—
Glu^j^	✓	✓	—
Safety evaluation	✓	✓	✓

a Not applicable.

b HRV: heart rate variability.

c PSG: polysomnography.

d MRI: magnetic resonance imaging.

e PSQI: Pittsburgh Sleep Quality Index.

f SF-36: 36-item Short Form Health Survey.

g ISI: Insomnia Severity Index.

h DBAS-16: 16-item Dysfunctional Beliefs and Attitudes About Sleep scale.

i GABA: γ-aminobutyric acid.

j Glu: glutamic acid.

### Participant Recruitment

Participants in this study will be recruited from the outpatient Department of Acupuncture and Moxibustion of our hospital, through posters placed on the hospital’s announcement board and other ways. The recruitment information will detail the research content, purpose, process, and corresponding contact information.

The participants will be asked to first contact the research assistant for preliminary screening. Participants who may be eligible will be registered, and a specialist will be appointed to check whether they meet the inclusion and exclusion criteria of this study (Textbox 1). Only patients who meet all the inclusion criteria and do not meet any exclusion criteria will be invited to participate in the study, and they will be given a detailed introduction to the specifics of this RCT. All participants are required to sign informed consent forms and provide comprehensive personal information.

Textbox 1. **Inclusion and exclusion criteria.**

**Inclusion criteria**

- Patients with symptoms in line with the diagnostic criteria of insomnia of liver depression and spleen deficiency type in traditional Chinese medicine
- Patients with symptoms in line with the western diagnostic criteria for insomnia [14]
- Male or female patients aged 18 to 40 years
- Patients with a disease course of more than 2 weeks

**Exclusion criteria (patients who meet any one of the following criteria will be excluded)**

- Patients with insomnia caused by a variety of organic diseases or neurological or psychiatric disorders
- Pregnant or lactating patients or those with a drug allergy
- Patients with more serious primary diseases such as cardiovascular and cerebrovascular diseases, gastrointestinal diseases, endocrine system diseases, or psychiatric disorders
- Patients with coagulation dysfunction and bleeding tendency

The baseline assessment will be performed by the same physician, and the results will be recorded in detail in the case report form. All participants will receive treatment for 4 weeks and undergo follow-up observation after treatment. The evaluation will be done at 3 time points: before treatment, after 4 weeks of treatment, and at 4 weeks after end of treatment. Each participant will receive a schedule of treatment dates and a schedule of follow-up appointments developed by research assistants who will be blinded to the grouping information.

In addition, all participants will be required to keep a sleep diary throughout the treatment and follow-up periods, recording the date of each insomnia episode, the length of time required to fall asleep, the number of times they wake up at night, the difficulty of falling asleep again, and the subjective feelings after waking up in the morning. Moreover, they will be asked to include sleep-related details as well as the name, dose, and time of use of other drugs (such as sleeping pills) during the trial and their own feelings after taking the drug.

### Ethical Considerations

The study was approved by the Medical Ethics Committee of the Third Affiliated Hospital of Zhejiang Chinese Medical University (approval no. ZSLL-KY-2024-032-01). If the main protocol needs to be modified, all members of the research team will participate in the process and submit the final modified protocol to the Medical Ethics Committee for review. This approval ensures that the study adheres to ethical standards and protects the rights and safety of participants. Patients and/or the public were involved in the design, conduct, reporting, or dissemination plans of this research. All participants are required to provide written informed consent to participate in this study. The consent process explicitly emphasizes the voluntary nature of participation and the right to withdraw at any time without penalty. To ensure privacy, all study data will be deidentified for analysis, with personal information stored separately under strict access controls. No financial compensation will be provided to participants.

### Randomization and Blinding

This is a single-blind RCT. In total, 96 patients with insomnia who meet the inclusion criteria will be randomly assigned (1:1) to the acupuncture group and control group. Statisticians who are blinded to the participants’ recruitment or intervention process will use the block randomization method to set the block size to 4 and generate a random distribution sequence. The assigned sequence will be hidden using serially numbered, opaque, sealed envelopes. After a participant has provided informed consent and completed the baseline assessment, the therapist will open the next serially numbered envelope in the presence of the participant to reveal the group assignment. Given the nature of acupuncture interventions, acupuncturists who perform the treatment cannot be blinded to group assignment. To ensure the integrity of the single-blind design, the following steps will be taken. Participants will not be informed of their specific group assignment but will be informed that they are receiving 1 of the 2 active acupuncture treatments for insomnia. Outcome evaluators and statisticians who perform data analysis will be blinded to group assignment. All data collection forms and electronic databases will use only participant identification codes and will not indicate group assignment. After all analyses have been completed, the allocation list will be securely maintained by the primary investigator until the database is locked. A total of 48 healthy volunteers without sleep disorders will be included as a nonrandomized observation control group without any intervention. The purpose of this group is to establish standardized physiological and questionnaire-based baseline values, which will be compared with the data of the patient groups. Given the nonrandomized allocation of healthy controls, statistical comparisons between patients and healthy controls would be considered to be observational in nature. To address the potential confounding factors, in appropriate cases, covariate adjustment and sensitivity analysis will be used to enhance causal inference to explain the differences between the patient groups and the healthy control group.

To ensure successful blinding, acupuncture will be performed for patients from both groups using several common approaches: skin sterilization with 75% alcohol; usage of disposable and sterile steel needles (1.5-inch needle, 0.25×40 mm); and insertion of needles into the skin to a depth of 10 to 15 mm, left for 30 minutes. Acupuncture treatment will be performed 3 times a week, and a total of 12 acupuncture or conventional acupuncture operations will be performed in 4 weeks. In addition, the external conditions such as the nursing process and the environment during the treatment will be kept consistent for the 2 groups of patients, so as to minimize the interference of additional factors on the results of the study.

### Interventions

#### Overview

All participants will receive acupuncture based on the Jing Bie theory or conventional acupuncture at a frequency of 3 times a week. To ensure the accuracy and consistency of the treatment administered, all acupuncture physicians participating in this study are required to have passed the clinical standard operation training in this subject and have the corresponding professional qualifications. Moreover, any possible adverse reactions during the study will be recorded in detail.

During the study period, the use of any prescription or nonprescription drugs for insomnia will be prohibited in principle. Participants who are required to use drugs for other diseases, which may affect the central nervous system, or have a severe insomnia problem will be processed and recorded as a “significant program deviation” or “withdrawal from the study” event. For those who do need medication, their complete medication information will be included in the subsequent statistical analysis as a key covariate. All participants are required to maintain a relatively stable work and rest schedule during the study period and to record information such as their bedtime, wake-up time, and occurrence of night awakening in a daily sleep diary. The researchers will review the diary every week to identify and guide individuals who experience excessive daytime sleepiness (>1 h) or significant changes in work and rest schedule (>2 h). Such behavioral changes will be included as covariates in the analysis.

#### Acupuncture Group

This group will include 48 patients. The patients will be placed in a supine position. Acupuncture will be performed bilaterally at Tianrong (SI17) points, with the needle inserted backward obliquely at 45±5° to a depth of 1.0 to 1.5 inches, reinforcing and reducing horizontally, and the needle insertion will be stopped when the patient feels a heat sensation in the auricle.

Acupuncture will be performed bilaterally at Tianyou (TE16) points, with the needle pointing toward the tip of the nose. The needle will be inserted to a depth of 0.5 to 1.0 inch using flat reinforcing and reducing maneuvers, until the sense of soreness and distension obviously stops; the needle will be retained for 30 minutes. The location of acupuncture points is shown in Table 2.

**Table 2.** Acupoint selection and stimulation parameters for the acupuncture group.

Acupoint	Location	Manipulation
Tianyou (TE16)	On the lateral side of the neck, located in the depression at the posterior border of the sternocleidomastoid muscle, level with the mandibular angle.	The needle is directed toward the tip of the nose, the insertion depth is 0.5‐1.0 inch, the reinforcing and reducing maneuvers are flat, needle insertion is stopped when soreness is obvious, and the needle is retained for 30 min.
Tianrong (SI17)	In the lateral aspect of the neck and in the depression on the anterior border of the sternocleidomastoid muscle and posterior to the mandibular angle	The needle is inserted backward obliquely at 45±5° to a depth of 1.0‐1.5 inches, with reinforcing and reducing maneuvers, and needle insertion is stopped when the patient feels a heat sensation in the auricle.

#### Control Group

This group will include 48 patients. The patients will be placed in a supine position. Acupuncture will be performed at Baihui (DU20), Sishencong (EX-HN1), bilateral Qimen (LR13), Zhangmen (LR14), Hegu (LR14), and Taichong (LR3), with reinforcing and reducing maneuvers. The needle insertion will be stopped when acid swelling is obvious, and the needle will be retained for 30 min. The location of acupuncture points is shown in Textbox 2.

Textbox 2. **Acupoint selection and stimulation parameters for the control group.**

**Acupoints and location**

- Baihui (DU20): on the head, 5 inches straight up the hairline or the midpoint of the line between the 2 ear tips
- Sishencong (EX-HN1): on the top of the head, a total of 4 points located 1 inch anterior, posterior, and bilateral to the Baihui
- Qimen (LR13): on the chest, in the sixth intercostal space, approximately 4 inches lateral to the anterior median line (midline) and level with the nipple.
- Zhangmen (LR14): on the lateral abdomen, below the free extremity of the 11th rib
- Hegu (LI4): on the dorsum of the hand, between the first and second metacarpals, at the midpoint of the radial side of the second metacarpal
- Taichong (LR3): on the dorsal side of the foot, in the posterior depression of the first metatarsal space

**Manipulation**

- Tonifying and reducing, until the soreness and distension obviously stops; the needle is retained for 30 minutes

#### Healthy Group

This group will include 48 healthy volunteers without sleep disorders, who will not undergo any treatment.

### Outcome Measures

#### Primary Outcome Measures

##### Sleep Quality Assessed Using the Pittsburgh Sleep Quality Index

The patients will be assessed using the Chinese version of the Pittsburgh Sleep Quality Index (PSQI) questionnaire at each observation point, and the clinical efficacy will be assessed. The questionnaire consists of 19 self-evaluation items and 5 other evaluation items. Only the 19 self-evaluation questions will be scored, which comprise 7 factors, each scored on a scale of 0 to 3 points (0: no difficulty, 3: serious difficulty). The sum of all factor scores ranges from 0 to 21, with a total score of 21 indicating serious difficulty in all respects. The reference values for adult sleep quality problems in China are as follows: PSQI ≥8 indicates poor sleep quality, whereas PSQI ≤7 indicates good sleep quality; the higher the score, the worse the sleep quality.

##### Quality of Life Assessed Using the 36-Item Short Form Health Survey

The patients will be evaluated using the 36-item Short Form Health Survey (SF-36) at each observation point. The SF-36 is a general scale developed by the American Medical Research Group to evaluate the quality of life. It is widely recognized and used worldwide. This scale has 8 dimensions to evaluate health-related quality of life, which belong to 2 categories of physical health and mental health, namely physiological function, role-physical, bodily pain, general health, vitality, social function, role-emotional, and mental health. In addition, the SF-36 scale includes another indicator, health change, which is used to evaluate health changes over the past year.

### Secondary Outcome Measures

#### Insomnia Severity Index

The patients will be evaluated using the Insomnia Severity Index (ISI) at each observation point. The degree of insomnia is determined according to the total score: a score between 0 and 7 indicates no clinically significant insomnia, a score between 8 and 14 indicates subthreshold insomnia, a score between 15 and 21 indicates clinical insomnia (moderate to severe), and a score between 22 and 28 indicates clinical insomnia (severe).

#### 16-Item Dysfunctional Beliefs and Attitudes About Sleep Scale Score

The items in the 16-item Dysfunctional Beliefs and Attitudes About Sleep scale (DBAS-16) comprise 4 factors: potential consequences of insomnia, worry about sleep, sleep expectations, and cognition of insomnia drugs. The lower the total score, the more likely it is that the participants’ cognition of sleep is biased.

#### γ-Aminobutyric Acid

γ-Aminobutyric acid (GABA) is used as a secondary outcome indicator for insomnia treatment, mainly based on its physiological role as a central inhibitory neurotransmitter. Studies have shown that the hyperarousal state of patients with insomnia is closely related to a decrease in GABA levels in brain regions such as the anterior cingulate gyrus and occipital cortex, and GABA concentration is negatively correlated with objective sleep indicators (such as awake time during sleep). Magnetic resonance spectroscopy can noninvasively and quantitatively determine the concentration of GABA in specific brain regions in vivo, thereby directly assessing whether the treatment improves sleep by enhancing the key mechanism of inhibitory neurotransmission.

#### Glutamic Acid

Glutamic acid (Glu) will be used as a secondary outcome indicator due to its key role in maintaining cortical excitability. Insomnia is often accompanied by excessive activation of the brain, which is related to abnormal activity of the glutamatergic system. Studies have shown that changes in the Glu levels in specific brain regions of patients with insomnia are associated with subjective sleep quality, anxiety, and anhedonia. Magnetic resonance spectroscopy can provide direct and quantitative information on Glu at the brain region level and thus will be used to verify whether the treatment restores the excitatory-inhibitory balance of the brain network by regulating excitatory neurotransmission.

### Indicators of Mechanism

#### Heart Rate Variability

The following HRV indices will be assessed: very LF (vLF), LF, HF, total power (TP), and LF/HF. Additionally, the parasympathetic nerve index (Log L×T) and sympathetic nerve index (Log L/T) will be calculated, where L denotes LF and T denotes TP, to evaluate ANS activity.

#### Polysomnography

The participants will be monitored by polysomnography at the TCM Diagnosis and Treatment Center of Sleep Disorders in the Department of Acupuncture and Moxibustion of our hospital after 4 weeks of treatment and at 4 weeks after the treatment ends. The monitoring was performed using a Philips polysomnography system (model: Alice 6). Positioning and interpretation will be performed according to Berry et al [15]. The changes in the sleep parameters of patients will be analyzed using the data collected by Sleepware G3 (Philips Respironics), including sleep efficiency, the proportion of each sleep stage, sleep latency, and other sleep indicators corresponding to other stages.

#### Magnetic Resonance Imaging

In this study, a GE Discovery MR750 1.5T MRI scanner will be used for imaging. During the test, the participant will be placed in a supine position, with their head held stable with a fixation frame. Moreover, the participant will be asked to wear earplugs to reduce stimulation due to equipment noise, so that the participant can relax as much as possible. First, a routine sequence axial scan will be performed to exclude organic lesions in the brain or the presence of artifacts; GABA and Glu experimental data will be collected again if there is no definite abnormality in the brain.

### Safety Evaluation

Adverse events during the study will be recorded and reported, and the patients will be treated in a timely and reasonable manner. The researchers will explain to the patients that they (or their families) are required to truthfully communicate the changes in their condition after treatment. The physicians will avoid guiding questions. During the observation of a curative effect, the researchers will pay attention to identify any adverse reactions.

Adverse events such as bleeding, hematoma, fainting, pain at the acupuncture site, and elevated blood pressure caused by acupuncture at any time will be recorded. Regardless of whether the adverse reactions or adverse events are related to the treatment method of this study, they will be recorded in detail. When these conditions occur, the patients will be recorded and treated properly in time until they completely return to normal, and the causes of adverse reactions will be analyzed.

### Data Management

The researchers will fill in the collected data into the case report forms according to the requirements of the research program. At the end of the study, the researchers will submit the case report forms of all patients included in this study to the data management center. These case report forms are required to be complete and signed. An internal data consistency check will be performed on all case report forms. Any inconsistencies identified will be logged, and a formal query will be generated and sent to the principal investigator for clarification and resolution.

### Sample Size and Statistical Methods

The main outcome measure of this study will be the total PSQI score at 4 weeks after treatment. The sample size was calculated on the basis of the expected difference between the test acupuncture group and the conventional acupuncture group in this index.

The expected effect size was determined as follows: studies have shown that acupuncture can significantly reduce PSQI scores compared with conventional therapy, with a combined mean difference of 2.0 to 3.5 points [16]. With reference to an RCT of similar design [17], we conservatively estimated that the mean difference (Δ) of PSQI between the 2 groups after treatment was 2.5 points, and the combined SD was approximately 4.0 points. According to this calculation, the standardized effect size is 0.625 (Δ/SD=2.5/4.0). Using G*Power software (version 3.1; Heinrich-Heine University Dusseldorf), setting α=.05 (bilateral) and test efficiency (1-β)=80%, according to a 1:1 distribution, the minimum sample size of each group was calculated to be 43. Considering a dropout rate of approximately 10%, 48 patients will be included in each group, and a total sample size of 96 patients was finally determined.

SPSS software (version 22.0; IBM Corp) will be used to analyze the collected data. All data will be expressed as mean (SD). For measurement data of the 2 groups, normally distributed data will be analyzed by independent 2-tailed *t* test and paired 2-tailed *t* test. If the data are not normally distributed, the Mann-Whitney rank sum test will be used. One-way ANOVA will be used for comparison between groups. Count data will be analyzed using a chi-square test, and rank data will be compared using the rank sum test and other analysis methods. A *P* value of ≤.05 will be considered statistically significant. To control for potential confounding, the use of concomitant medications and measures derived from sleep diaries will be incorporated as covariates into a generalized linear model for sensitivity analysis of the primary outcomes.

### Final Report and Publication

After the end of the study, the main researchers will take the lead to cooperate with the researchers at each clinical department to write the research summary report, and the researchers at each clinical department will sign the research summary report. The research report will include a description of the research purpose, the methods used in the research, and the results and conclusions.

## Results

This study was funded in November 2023. Recruitment and data collection began in January 2024. The results will be shared with participants, researchers, and the public via conferences, publications, and online platforms. The findings will be submitted to a peer-reviewed journal. Participant recruitment is underway and will continue until the target sample size of 96 patients with primary insomnia of liver depression and spleen deficiency type and 48 healthy controls is reached. Participant recruitment is scheduled to be completed by the end of 2026. Each enrolled patient will undergo the 4-week intervention followed by a 4-week follow-up period as per the study protocol. As of December 2025, a total of 40 participants have been enrolled, of whom 4 have withdrawn. Data collection is expected to conclude by the end of 2026, with full data analysis and manuscript preparation completed by summer 2027.

## Discussion

### Study Rationale

In this study, an RCT was designed to evaluate the efficacy of acupuncture at specific meridians and the acupoints SI17 and TE16 on patients with primary insomnia of liver depression and spleen deficiency type and to conduct a parallel assessment of ANS regulation using HRV and neurochemical indicators.

The selection of the cervical acupoints SI17 and TE16 is based on the classical meridian divergence (Jing Bie) theory of TCM [5]. According to “Lingshu · Jing Bie,” although SI17 belongs to the small intestine meridian, it is located at the intersection of the liver and gallbladder meridians. Therefore, acupuncture at SI17 can soothe and regulate the liver and gallbladder qi. Although TE16 belongs to the triple energizer meridian, it is located at the intersection of the triple energizer, pericardium, and gallbladder meridians. Acupuncture at TE16 can regulate the qi of the Shaoyang meridian, relieve the stagnation of the upper energizer and tranquilize the mind, and provide a theoretical basis for related diseases. In TCM, the neck is a key region where multiple yang meridians converge [18]. As pivotal acupoints in the neck, SI17 and TE16 are essential for regulating the mind and balancing yin and yang, and their clinical values stem from the deep regulation of ying-wei qi and blood by the hand Taiyang and hand Shaoyang meridians. Specifically, the Tianrong point follows the path of “entering the armpit and walking the heart” by the hand Taiyang meridian, which has the effect of dredging the neck qi and blood and calming the heart and nerves. Its anatomical deep layer corresponds to the cervical sympathetic ganglion, which can inhibit the excessive excitement of the sympathetic nerve. The Tianyou acupoint, with the function of the hand Shaoyang meridian “scattered in the chest,” can regulate the triple energizer qi, straighten out the upper energizer, and affect the activity of the vagus nerve. According to the “Lingshu · Jing Bie,” these divergences “branch from the main channels, enter the body cavity, link exterior and interior, and harmonize *ying* (nutritive) and *wei* (defensive) energies.” The yang meridians are different from the original meridians, and the yin meridians are different from the yang meridians in the exterior and interior, which strengthens the close relationship between the limbs and the zang-fu organs, head, and face. In TCM, insomnia of the liver depression and spleen deficiency type is considered to stem from disrupted qi flow and impaired balance between ying and wei [19,20]. The mechanism of acupuncture involves regulating the relationship between yin and yang, the unity of opposites, to restore the harmonious flow and balance of qi in the whole body [21]. On the basis of this principle, specific acupoints on the neck are stimulated to dredge the Shaoyang meridian and reconcile ying and wei so as to promote yang qi into yin and support natural sleep. This follows the classical TCM principle that “acupuncture treats areas along the pathway of the meridian.”

Notably, this traditional rationale aligns with modern neuroanatomical insights. The cervical regions where these meridian divergences run are anatomically close to major sympathetic ganglia, such as the stellate ganglion [22,23]. Therefore, acupuncture at SI17 and TE16 may modulate local sympathetic nerve activity, in a way similar to SGB—a procedure known to influence autonomic function [24-26]. This integrated view connects the TCM concepts of qi regulation and ying*-*wei harmony with the modern understanding of ANS balance, offering a coherent mechanistic framework for acupuncture.

### Proposed Mechanism and Empirical Testing

The proposed mechanism can be traced through specific physiological pathways. The anatomical proximity of these points to the cervical sympathetic chain means that stimulation may send signals through the spinal cord to higher brain regions that regulate autonomic function, such as the brain stem and hypothalamus [27,28]. This influence on the central nervous system can rebalance the sympathetic and parasympathetic activity, a change measurable through HRV [29-32]. Thus, HRV provides an objective link between the TCM idea of restored ying-wei harmony and modern metrics of ANS balance. Beyond this neural pathway, the intervention likely also involves neuroendocrine adjustments [33-35]. Chronic insomnia and the TCM pattern of liver qi stagnation are both associated with overactivity of the body’s stress response system, the hypothalamic-pituitary-adrenal axis [36-38]. By mitigating excessive sympathetic drive, acupuncture may help calm the hypothalamic-pituitary-adrenal axis hyperactivity [39], potentially leading to lower cortisol levels and a more sleep-conducive internal milieu [40-42]. In summary, the treatment is hypothesized to work through a convergent network, modulating both neural excitability and endocrine function to address the multifaceted pathophysiology of insomnia.

To empirically test this mechanistic model, our study employs a multimethod assessment strategy. In addition to standard patient-reported outcomes (PSQI and ISI), we will measure HRV to directly quantify ANS changes [43]. We will also analyze the levels of key neurotransmitters—GABA and Glu. We hypothesize that successful treatment will be correlated with a shift toward a less excited central nervous system state, reflected in an increased GABA to glutamate ratio [18]. This combined biochemical and physiological evidence aims to clarify how stimulation at these specific points translates into clinical improvement, thereby strengthening the scientific rationale for this targeted acupuncture protocol.

This study incorporates a parallel cohort of healthy controls to establish normative baselines for HRV and relevant neurochemical markers. These reference values will enable the quantitative assessment of the severity of ANS dysfunction in patients with insomnia and provide a benchmark for evaluating postintervention recovery. This comparative design strengthens the robustness of the findings and supports a multidimensional evaluation of treatment efficacy.

To comprehensively capture both objective and subjective dimensions of sleep and autonomic function, the primary outcome indicators include the PSQI and SF-36. The secondary outcome indicators include the ISI and DBAS-16 as well as GABA and Glu levels. These 2 neurotransmitters were selected as biomarkers because they play an important role in sleep-wake regulation and the high arousal state characterized by primary insomnia. Assessing these neurochemicals will help to elucidate the mechanisms underlying treatment response.

Methodologically, the trial employs a single-blind, randomized design with block randomization to minimize selection bias. The sample size was determined by an a priori power calculation to ensure adequate statistical power, and all acupuncture procedures will be performed adhering to a strict standardized protocol to maintain treatment fidelity across participants. Focusing exclusively on patients with the liver depression-spleen deficiency pattern will reduce clinical heterogeneity, allowing a more precise investigation of acupuncture mechanisms within this defined TCM syndrome framework.

### Limitations

Several limitations warrant consideration. Achieving complete blinding in acupuncture trials remains inherently challenging, and the participant age range (18‐40 y) may constrain the generalizability of the findings to older populations. Nevertheless, the protocol aligns with the CONSORT (Consolidated Standards of Reporting Trials) guidelines through structured data collection, systematic adverse event monitoring, and prespecified statistical analysis—features that collectively uphold the internal validity and interpretability of the study outcomes.

This study has some limitations. First, the scope of sample selection is limited. The inclusion criteria limited participant age to 18 to 40 years and the course of the disease to more than 2 weeks. Although a narrow age range is conducive to controlling confounding age factors, it cannot represent the situation of patients with insomnia in other age groups, which limits the universality of the research results and may lead to age-related bias in the conclusions. Second, there are limitations in the blinding design. Due to the significant difference between the intervention strategies of the Jing Bie theory acupuncture group and the traditional acupuncture group, the blinding protocol for acupuncture physicians faces challenges in practice. Therefore, this study adopts a single-blind design. Although this may, in theory, introduce operator bias, several measures will be taken to maintain methodological rigor: the acupuncture physician will follow strict standardized operating procedures; the outcome evaluators and data analysts will remain blinded to the grouping; and the participants will know only that they received active acupuncture intervention and will not know the specific grouping. These measures aim to minimize potential bias while maintaining the authenticity of clinical practice. Future studies may consider using sham acupuncture or noninvasive needles in the control group to achieve double blinding, although such methods also have inherent limitations in simulating real acupuncture effects.

In summary, this study will explore a new path for acupuncture treatment of insomnia based on the theory of Jing Bie and establish acupoint selection (targeting cervical regions to regulate autonomic function) and evaluation (integrated comprehensive evaluation program using PSQI, HRV, and neurochemical biomarkers), providing a reference for subsequent research. However, the limitations of sample representativeness and blinding design suggest that future research needs to be improved in terms of expanding the age range of the sample and optimizing the strategy of implementation of blinding, so as to further verify and expand the findings of this study.

### Conclusion

This study is expected to fill the following evidence gaps.

#### Efficacy Evidence of Specific Syndromes

At present, most of the studies on acupuncture treatment of insomnia do not strictly distinguish TCM syndromes. This study will focus on insomnia with liver depression and spleen deficiency and provide high-quality RCT evidence for specific syndromes.

#### Mechanism of ANS Regulation

Previous studies have not sufficiently discussed the mechanism of acupuncture regulating autonomic nerve function. This study will provide a more comprehensive explanation for the mechanism of acupuncture in improving insomnia through an in-depth analysis of HRV and other indicators.

#### Long-Term Efficacy Evaluation

Most acupuncture studies only focus on short-term efficacy. This study will use a 4-week follow-up to evaluate the long-term effect of acupuncture and provide a more reliable basis for clinical application.

#### Changes in Neurotransmitter Levels

In this study, GABA and Glu levels will be determined to fill the evidence gap of acupuncture regulation of neurotransmitters in patients with insomnia and to provide a new direction for the neurobiological mechanism of acupuncture treatment of insomnia.

## Supplementary material

10.2196/84122 Checklist 1 SPIRIT checklist.

## Abbreviations

ANS: autonomic nervous system
CONSORT: Consolidated Standards of Reporting Trials
DBAS-16: 16-item Dysfunctional Beliefs and Attitudes About Sleep scale
Glu: glutamic acid
HF: high-frequency power
HRV: heart rate variability
ISI: Insomnia Severity Index
LF: low-frequency power
Log L/T: sympathetic nerve index
Log L×T: parasympathetic nerve index
PSQI: Pittsburgh Sleep Quality Index
RCT: randomized controlled trial
SF-36: 36-item Short Form Health Survey
SGB: stellate ganglion block
SPIRIT: Standard Protocol Items: Recommendations for Interventional Trials
TCM: traditional Chinese medicine
TP: total power
vLF: very low-frequency power
